# Optimal Pest Control Strategies with Cost-effectiveness Analysis

**DOI:** 10.1155/2021/6630193

**Published:** 2021-04-21

**Authors:** Ihza Rizkia Fitri, Farida Hanum, Ali Kusnanto, Toni Bakhtiar

**Affiliations:** Department of Mathematics, Faculty of Mathematics and Natural Sciences, IPB University, Bogor 16680, Indonesia

## Abstract

Pest and plant diseases cause damages and economic losses, threatening food security and ecosystem services. Thus, proper pest management is indispensable to mitigate the risk of losses. The risk of environmental hazards induced by toxic chemicals alongside the rapid development of chemical resistance by insects entails more resilient, sustainable, and ecologically sound approaches to chemical methods of control. This study evaluates the application of three dynamical measures of controls, namely, green insecticide, mating disruption, and the removal of infected plants, in controlling pest insects. A model was built to describe the interaction between plants and insects as well as the circulation of the pathogen. Optimal control measures are sought in such a way they maximize the healthy plant density jointly with the pests' density under the lowest possible control efforts. Our simulation study shows that all strategies succeed in controlling the insects. However, a cost-effectiveness analysis suggests that a strategy with two measures of green insecticide and plant removal is the most cost-effective, followed by one which applies all control measures. The best strategy projects the decrease of potential loss from 65.36% to 6.12%.

## 1. Introduction

For decades, pests and plant diseases have been challenging our food security systems as they cause yield and quality degradation of crops production. Limited data available suggest an annual loss of 18–20% in crop production worth USD470 billion due to arthropods worldwide [1]. It was estimated by [2] that yield losses of major food crops comprise rice losses of 30%, maize losses of 22.5%, wheat losses of 21.5%, soybean losses of 21.4%, and potato losses of 17.2% on average globally. This burden is compounded by the threat of global warming as a warmer climate will accelerate the metabolic rate of pest insects and the insects' food consumption rate, leading to an explosion of pest insects' population particularly in elsewhere of nontropical regions. All climate models project an increase of 10–25% of economic losses per degree C of global temperature warming [3].

It is commonly known that the use of universal chemical control such as conventional pesticides brings environmental drawbacks. Knowing that, integrated pest management (IPM) is developed. It is a framework of pest management and strategies in such a way that it minimizes overall economic, health, and environmental risks [4, 5]. From an extended standpoint, the ecological and environmental in nature of IPM has been revisited, for instance by [6], to include sustainability, business, and management aspects and strengthen the importance of research and its implementation. In this context, reducing risks of crop losses due to pests and plant diseases has become one of the main concerns of IPM. There is thus a great need to find efficient and sustainable pest management strategies.

The application of selective pest control based on pesticide selectivity tests is recommended in preserving these natural enemies as biological control instruments [7]. Selective pesticides promote the use of more satisfactory chemical insecticides, which in addition to being effective and satisfy certain criteria, they must not pose immediate or long-term risks to crops ecosystems. Such kind pesticides include the application of the novel chemistry, nonsteroidal ecdysone agonists bisacylhydrazine (BAH) compounds, which have been commercialized to specifically manage coleopteran, lepidopteran, and dipteran larvae [8, 9]. BAH insecticides such as tebufenozide and halofenozide have been identified to display highly selective toxicity to target pest insects by disrupting the typical physiology of larvae growth-stimulating abnormality and kill larvae in the process. BAH compounds have been known for their use as a class of green insecticides [10].

Another type of control is exploiting the sex pheromones which have a prominent and deep-rooted role in pest management strategies due to their nondamaging impact on the environment [11]. Mating disruption is a direct control technique by the release of synthetic sex pheromones in a much higher amount than a female can produce to interfere with the mate finding process. The behavioural response of male insects is disrupted by their exposure to the abundance of synthetic sex pheromones, leading to the reduction of eggs laying and larvae incidence [12, 13]. In application, the deployment of synthetic sex pheromone in the mating disruption program can be undertaken by using passive dispensers [14], or even through an aerosol delivery system, a more effective system as it can be applied at far lower density (2–5 units per hectare) than a passive dispenser, and it can be operated at certain periods following the active period of the insects [15]. Pest control by using mating disruption technique has commonly been adopted to mediate the behaviour of a different type of insects on various type of plants, e.g., white grub beetle in sugarcane [16], stem borer and leaf folder in rice [17], the light brown apple moth in pine forest [18], tortricid moth in apple and vineyards [19], and the control of coleopteran and fruit flies [11].

Unfortunately, the application of more ecofriendly pest controls may come with a higher cost. Thus, in this study, we demonstrated an optimal control approach to find the best combination of pest insect control that could prevent crop loss due to physical damage or crop vector-borne plant diseases with the cheapest cost. We proposed a model with eleven compartments to describe the life cycle of pest insect, the size of plant density, and the circulation of the pathogen. We then conducted a cost-effectiveness analysis to compare strategies incorporating different control combinations.

## 2. Modelling of Pest-Plant Interaction

By the advancement of ecological and biophysical knowledge as well as computational methods, the needs of more complex, experimental data-driven models are emphasized in integrated pest control strategies for gaining more accuracy and more applicability to field situations than simpler analytical models [20, 21]. Among all, Jung et al. [22] modelled the potential distribution of invasive pest spotted lanternfly by exploiting climate data, temperature, and moisture indices and environmental stress index using climate and population modelling software CLIMEX. Such kind of dynamic population models can then be extended by integrating pests and crops models [23, 24]. In this development, the existence of equilibria and their stability analysis [25–28], the basic reproduction number [24], the bifurcation analysis [29], and the impact of some key parameters on the transmission dynamics of disease [5, 24] are often the focus of study. In [24], the framework of deterministic and stochastic modelling revealed that the parameters on disease transmission are crucial to the dynamical process, as a small perturbation in these parameters contributes detrimental effects in the infective populations. Another pest control model considering the stochastic effect was carried out by [30].

Another direction of research in this field is related to the development of pest-crop models which enable us to intervene in the dynamic interaction among components utilizing control variables such as the use of pesticides [31], the release of sterile insects [32, 33], the release of natural predators [34], the application of mating disruption [35], the use of mass trapping [36], and the removal of infected host plants [37]. In this framework, determining the optimal control strategies for a certain performance criterion is often the research objective. A variant of the pollution emission model was developed by [38] to simulate the residual and delayed effects of spraying pesticides on pests incorporating the stage structure of population and birth pulse. A mathematical model of SCIR (susceptible-cryptic-infected-removed) was developed by [39] to evaluate the effectiveness of several host plants removal strategies toward the spread of citrus canker. The removal strategies include the risk-based control, variable radius strategy, and constant radius strategy. It was revealed that removal of host plants suspected to cause a higher number of infections in the remaining population, i.e., risk-based control strategy outperforms radius-based strategies and is robust to parameters changes of disease spread. A dynamical model of biological pest insects control using the sterile insect technique was developed by [25], incorporating the interaction between pest insects and crops population. It is shown that the sterile insect release rate plays pivotal roles and provides a significant influence in controlling fertile pests' density in the population as well as in determining the existence and extinction of the crops population. Barclay and Judd [35] developed a daily events model to evaluate three different mechanisms of mating disruption, namely, confusion of males, emigration of males before mating, and false trails due to competition with female pheromone trails. Kang et al. [40] exploited a hybrid dynamical model of two competing pests and their natural predator to determine the optimal control strategies regarding the natural predator choice, the release time of natural predator, the dose and timing of insecticide spraying, and the killing rate of the pesticide.

The optimal control approach is pretty common to investigate the best control strategies. Interaction of pest-predator-virus was formulated in an optimal control model by [41], where the control objective was to determine the rates of spraying of chemical and viral pesticides such that the pest population was kept under the injury level and the biomass of crops reached the highest possible level. The optimization task was undertaken for the maximization of a certain profit function. Another example is the study by Kar et al. [42] which investigated the optimal use of pesticides to reduce the density of susceptible and infected pests in a pest-predator-virus model. The combination use of bio and chemical pesticides in pest management of *Jatropha curcas* was studied by [31], employing an optimal control framework to balance environmental loss and economic costs of controls.

## 3. Pest Insects Control Model

Our model consists of two interacting populations, namely, pest insects' population and plant's population, usually crops. In constructing our pest control model, we take into consideration the life cycle of insects [36], the prey-predator interaction between insects and plants [25], and the process of pathogen transmission among insects and plants [29]. We propose a new model of pest insects' control which comprises eleven disjoint compartments to represent the interaction between insects, plants, and the circulation of the pathogen. We denote *L*_*I*_(*t*), *M*_*I*_(*t*), *Y*_*I*_(*t*), and *F*_*I*_(*t*) as the size of infectious larva, infectious males, the infectious unfertilised females, and the infectious fertilised females at time *t*, respectively. And we denote by *L*_*S*_(*t*), *M*_*S*_(*t*), *Y*_*S*_(*t*), and *F*_*S*_(*t*) the size of noninfectious larvae, noninfectious males, the noninfectious unfertilised females, and the noninfectious fertilised females at time *t*, respectively. The plant's population is divided into two classes, namely, susceptible and infected plants. Their sizes at time *t* are represented by *S*(*t*) and *I*(*t*), respectively. One additional compartment is added to represent the synthetic sex pheromone in the system and is measured as the number of fake female insects (*Y*_*f*_).

### 3.1. Assumptions and Compartmental Model

There is an immense diversity of interactions between plants and other species including pest insects. The following behavioural and biological assumptions are implemented in developing our model.

(1) The insects follow two development phases: larval and adult phases. The larval phase includes the growing of eggs, larvae, and pupae, while the adult phase relates to the growth of male and female insects. To some degree, this assumption is similar to that used by [36, 38].
(2) The population of larva grows logistically with the intrinsic egg-laying rate *b*_1_ and environmental carrying capacity depends on the plant, since insects mostly lay their eggs on the host plants for food resources [43]. The number of larvae that can be supported by one unit of plants weight per unit area is *c*.
(3) Larvae will grow to be mature susceptible insects at the rate of *v*_*L*_ with a constant female-to-male ratio of *r*
(4) Unfertilised females will go through the mating process with the rate of *v*_*Y*_, and the probability of a single unfertilised female to be mated is given by *ρ* in (1). If the number of males is less than that of females (male scarcity), then *ρ* is expressed as the male-to-female ratio, i.e., the sex ratio. Otherwise, if the number of males is large enough to mate with all unfertilised females (male abundance), then the probability of a single female to be mated is 1.
  (1) $$\begin{array}{c} \rho =\min\left\{1,\frac{\gamma \left(M_{S}+M_{I}\right)}{Y_{S}+Y_{I}+Y_{f}}\right\}. \end{array}$$
The parameter *γ* is the number of females that can be fertilised by a single male, and *v*_*Y*_*ρ* denotes the transfer rate from unfertilised class to fertilised class. By (1), we extend the expression used in [36] as adult insects differentiate into susceptible and infectious.
(5) It is assumed that a fertilised female can again become unfertilised at a rate of *δ*
(6) Generally, two interactions between insects and plants may happen
  (a) Pest insects will feed on plants with the rate of consumption in a day denoted by *η*_*j*_ for *j* ∈ {*L*, *M*, *Y*} which cause physical plant losses due to consumption and is quantified to the mortality rate of plants and with Holling type II response function [18].
  (b) Pathogen transfer in this ecology usually happens via bites by insects to plants and the consumption process, the most common way to transfer pathogen via the stylet of the insects [44]. The pathogen such as virus is transferred from infectious pest insects to susceptible plants with an infection rate of *α*_1,*a*_ for larva, *α*_1,*M*_ for male insects, and *α*_1,*Y*_ for the female insect. The pathogen such as virus is transferred from infected plants to susceptible pest insects with the rate of *α*_2,*L*_ for larva, *α*_2,*M*_ for males, and *α*_2,*Y*_ for females, since the pathogen transfer happens following the consumption activity. As for the species used in this work (*Planococcus ficus*), the males do not feed when they are adult due to their short life span, and their larvae also have different physical features [45]. In the case of many lepidopterans, their larvae are the biggest threat to agriculture [46].
(7) Physical damage and pathogen transmission are assumed to be able to happen simultaneously. Examples of this phenomenon include brown plant-hopper that damages rice paddy [47] and even insects with biting and chewing mouth parts [48].
(8) Plant-to-plant pathogen transmission is possible with the rate of infection of *α*_3_

**Figure 1:**
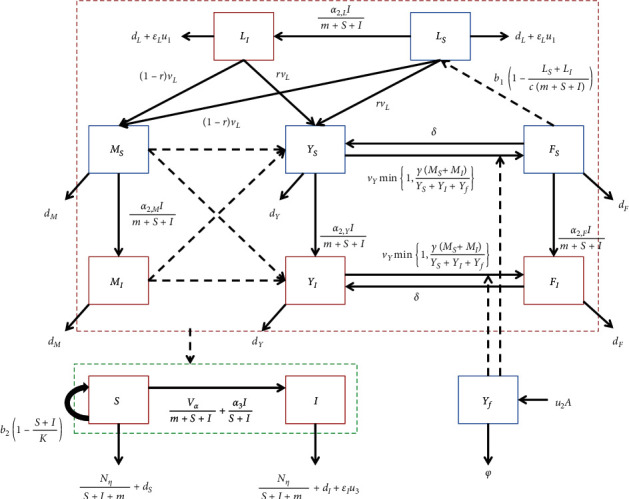
Compartmental diagram of the pest control model which consists of three population blocks: noninfectious insects, infectious insects, and plants.

Based on the abovementioned assumptions, we construct a compartmental model of plant-pest interaction dynamics as depicted in Figure 1. This compartmental model consists of nine classes of the population. The black solid arched lines represent the motion of the population transfer, while the black dashed lines represent the interactions between subpopulation which causes population transfer.

### 3.2. Mathematical Model

From the compartmental diagram depicted in Figure 1, the equations of motion among compartments, which show the interdependence between insects and plants populations, are represented by the following set of ordinary nonlinear differential equations:

(2) $$\begin{array}{c} {\dot{L}}_{S}=b_{1}\left(F_{S}+F_{I}\right)\left(1-\frac{L_{S}+L_{I}}{c\left(m+S+I\right)}\right)-\left(v_{L}+d_{L}+\frac{\alpha_{2,L}I}{m+S+I}+\varepsilon_{L}u_{1}\right)L_{S}, \end{array}$$

(3) $$\begin{array}{c} {\dot{L}}_{I}=\frac{\alpha_{2,L}IL_{S}}{m+S+I}-\left(v_{L}+d_{L}+\varepsilon_{L}u_{1}\right)L_{I}, \end{array}$$

(4) $$\begin{array}{c} {\dot{M}}_{S}=\left(1-r\right)v_{L}L_{S}-\left(\frac{\alpha_{2,M}I}{m+S+I}+d_{M}\right)M_{S}, \end{array}$$

(5) $$\begin{array}{c} {\dot{M}}_{I}=\left(1-r\right)v_{L}L_{I}+\frac{\alpha_{2,M}IM_{S}}{m+S+I}-d_{M}M_{I}, \end{array}$$

(6) $$\begin{array}{c} {\dot{Y}}_{S}=rv_{L}L_{S}+\delta F_{S}-\left(v_{Y}\rho +\frac{\alpha_{2,Y}I}{m+S+I}+d_{Y}\right)Y_{S}, \end{array}$$

(7) $$\begin{array}{c} {\dot{Y}}_{v}=rv_{L}L_{I}+\frac{\alpha_{2,Y}IY_{S}}{m+S+I}+\delta F_{I}-\left(v_{Y}\rho +d_{Y}\right)Y_{I}, \end{array}$$

(8) $$\begin{array}{c} {\dot{F}}_{S}=v_{Y}\rho Y_{S}-\left(\frac{\alpha_{2,Y}I}{m+S+I}+\delta +d_{F}\right)F_{S}, \end{array}$$

(9) $$\begin{array}{c} {\dot{F}}_{I}=v_{Y}\rho Y_{I}+\frac{\alpha_{2,Y}IF_{S}}{m+S+I}-\left(\delta +d_{F}\right)F_{I}, \end{array}$$

(10) $$\begin{array}{c} \dot{S}=b_{2}S\left(1-\frac{S+I}{K}\right)-\left(\frac{V_{\alpha }+N_{\eta }}{m+S+I}+\frac{\alpha_{3}I}{S+I}+d_{S}\right)S, \end{array}$$

(11) $$\begin{array}{c} \dot{I}=\left(\frac{V_{\alpha }}{m+S+I}+\frac{\alpha_{3}I}{S+I}\right)S-\left(\frac{N_{\eta }}{m+S+I}+d_{I}+\varepsilon_{I}u_{3}\right)I, \end{array}$$

(12) $$\begin{array}{c} {\dot{Y}}_{f}=Au_{2}-\phi Y_{f}, \end{array}$$

with following nonnegative initial conditions apply

(13) $$\begin{array}{c} L_{S}\left(0\right)=L_{S}^{0},\\ L_{I}\left(0\right)=L_{I}^{0},\\ M_{S}\left(0\right)=M_{S}^{0},\\ M_{I}\left(0\right)=M_{I}^{0},\\ Y_{S}\left(0\right)=Y_{S}^{0},\\ Y_{I}\left(0\right)=Y_{I}^{0},\\ F_{S}\left(0\right)=F_{S}^{0},\\ F_{I}\left(0\right)=F_{I}^{0},\\ S\left(0\right)=S^{0},\\ I\left(0\right)=I^{0},\\ Y_{f}\left(0\right)=Y_{f}^{0}. \end{array}$$

In (10) and (11), *V*_*α*_ and *N*_*η*_ are defined as follows:

(14) $$\begin{array}{c} V_{\alpha }=\alpha_{1,L}L_{v}+\alpha_{1,M}M_{v}+\alpha_{1,Y}\left(Y_{v}+F_{v}\right),\\ N_{\eta }=\eta_{L}\left(L+L_{v}\right)+\eta_{M}\left(M+M_{v}\right)+\eta_{Y}\left(Y+Y_{v}+F+F_{v}\right). \end{array}$$

Due to the limited number of plants to be consumed, the interactions between adult insects and plants are of predator-prey Holling type II with half-saturation constant whose intake rate for susceptible and infected plants consumption are, respectively, given by *f*(*S*) and *g*(*I*), where

(15) $$\begin{array}{c} f\left(S\right)≔\frac{\eta_{j}S}{m+S+I},\\ g\left(I\right)≔\frac{\eta_{j}I}{m+S+I}. \end{array}$$

By the existence of the mating probability *ρ*, the model in fact will switch between two environments according to the value of *ρ* given in (1). If the number of male insects is less than that of female insects, i.e., *γ*(*M*_*S*_+*M*_*I*_) < *Y*_*S*_+*Y*_*I*_+*Y*_*f*_, then equations (5)–(8) are, respectively, replaced by

(16) $$\begin{array}{c} {\dot{Y}}_{S}=rv_{L}L_{S}+\delta F_{S}-\left(v_{Y}\frac{\gamma \left(M_{S}+M_{I}\right)}{Y_{S}+Y_{I}+Y_{f}}+\frac{\alpha_{2,Y}I}{m+S+I}+d_{Y}\right)Y_{S},\\ {\dot{Y}}_{I}=rv_{L}L_{I}+\frac{\alpha_{2,Y}IY_{S}}{m+S+I}+\delta F_{S}-\left(v_{Y}\frac{\gamma \left(M_{S}+M_{I}\right)}{Y_{S}+Y_{I}+Y_{f}}+d_{Y}\right)Y_{I},\\ {\dot{F}}_{S}=v_{Y}\frac{\gamma \left(M_{S}+M_{I}\right)}{Y_{S}+Y_{I}+Y_{f}}Y_{S}-\left(\frac{\alpha_{2,Y}I}{m+S+I}+\delta +d_{F}\right)F_{S},\\ {\dot{F}}_{I}=v_{Y}\frac{\gamma \left(M_{S}+M_{I}\right)}{Y_{S}+Y_{I}+Y_{f}}Y_{v}+\frac{\alpha_{2,Y}IF_{S}}{m+S+I}-\left(\delta +d_{F}\right)F_{I}. \end{array}$$

Otherwise, if the number of male insects is greater than that of female insects *γ*(*M*_*S*_+*M*_*I*_) ≥ *Y*_*S*_+*Y*_*I*_+*Y*_*f*_, which leads to *ρ*(*t*)=1, then equations (5)–(8) are, respectively, replaced by

(17) $$\begin{array}{c} {\dot{Y}}_{S}=rv_{L}L_{S}+\delta F_{S}-\left(v_{Y}+\frac{\alpha_{2,Y}I}{m+S+I}+d_{Y}\right)Y_{S},\\ {\dot{Y}}_{I}=rv_{L}L_{I}+\frac{\alpha_{2,Y}IY_{S}}{m+S+I}+\delta F_{I}-\left(v_{Y}+d_{Y}\right)Y_{I},\\ {\dot{F}}_{S}=v_{Y}Y_{S}-\left(\frac{\alpha_{2,Y}I}{m+S+I}+\delta +d_{F}\right)F_{S},\\ {\dot{F}}_{I}=v_{Y}Y_{I}+\frac{\alpha_{2,Y}IF_{S}}{m+S+I}-\left(\delta +d_{F}\right)F_{I}. \end{array}$$

The former situation is referred to as male scarcity, and the latter is regarded as male abundance. Both situations are crucial in the insect reproduction process as sex ratio imbalance may influence the male-male competition over mating [49].

### 3.3. Control Instruments

Our model in (2)–(10) is equipped with three controls, namely, the use of green insecticide (*u*_1_), the application of synthetic sex pheromone for mating disruption (*u*_2_), and the removal of infected plants (*u*_3_). It is assumed that green insecticide is applied to inhibit larval growth and kill them in the process. Thus, the effect of green insecticide is administered only at the larval compartment *s* in (2) and (3). The control variable *u*_1_(*t*) is then defined as the proportion of the larval population that green insecticide is applied to at time *t*. The release of synthetic sex pheromones increases the size of the fake female (*Y*_*f*_) population which then indirectly reduces mating success rate. The effectiveness of mating disruption is specified by the amounts of active component in addition to the type of dispensers and the frequency of spraying [50]. We define by *u*_2_(*t*) the proportion of the maximum number of fake females released at time *t*. The third control is the plant removal and its side-work such as burning and burying which also require considerable labour and resources for collection, identification, and analysis. Examples of this action include the injection of herbicide (glyphosate) to kill all bananas infected by black Sigatoka and the thorough destruction of all apple trees with scab and the cutting back to basal dormant buds of any adjacent asymptomatic plants [37]. Another example is the mat uprooting in *Xanthomonas* wilt of banana [51]. We define by *u*_3_(*t*) the proportion of infected plant removed from the population at time *t*.

Since all control variables are related to proportions, then bounded control policies must be implemented, i.e., the controls are constrained within the bounds of

(18) $$\begin{array}{c} 0\leq u_{1}\left(t\right)\leq 1,\\ 0\leq u_{2}\left(t\right)\leq 1,\\ 0\leq u_{3}\left(t\right)\leq 1, \end{array}$$

for all *t* ∈ [0, *T*], where *T* is the length of the control period. We also assume that each control has its effectiveness denoted by *ε*_1_, *ε*_2_, and *ε*_2_, respectively, with *ε*_*i*_ ∈ [0,1] for *i*=1,2,3. For further analysis, we denote by *u* the vector of control variables, i.e., *u*(*t*)=(*u*_1_(*t*),*u*_2_(*t*),*u*_3_(*t*))^*T*^ and by *𝕌* the set of all admissible controls given by

(19) $$\begin{array}{c} U=\left\{u|u_{i}\left(t\right)\text{ is Lebesque measurable in }\left[0,T\right],u_{i}\left(t\right)\in \left[0,1\right],t\in \left[0,T\right]\right\}. \end{array}$$

## 4. Control Problem and Its Optimality Conditions

We describe in the previous section the dynamical of disease transmission within the interaction between pests and plants. We have already furnished the model with three biological control instruments, namely, the use of BAH green insecticide to reduce larval population, the deployment of synthetic sex pheromone to disrupt the mating process, and the removal of infected plants to hamper disease spread. Our main control objective is to maximize the size of healthy plants biomass under the lowest possible control efforts. The following performance index is then introduced:

(20) $$\begin{array}{c} J\left(u\right)=BS\left(T\right)-\int_{0}^{T}\left(C_{0}N\left(t\right)+C_{1}u_{1}^{2}\left(t\right)+C_{2}u_{2}^{2}\left(t\right)+C_{3}u_{3}^{2}\left(t\right)\right)\text{d}t. \end{array}$$

The first term in (20) accounts for the size of healthy plants, while the second term represents the size of the insect population; then, the rest represents the total of control efforts, which can indirectly be seen as the cost for applying control. Here, we assume that the control efforts are nonlinear (quadratic form). Thus, the maximal value of *J* can be attained by the maximization of *S*(*T*) which is the size of the plant at the end of the control period jointly with minimisation of *C*_0_*N*(*t*) and ∑_*i*=1_^3^*C*_*i*_*u*_*i*_^2^(*t*) for *N*(*t*)=*L*_*S*_(*t*)+*L*_*I*_(*t*)+*M*_*S*_(*t*)+*M*_*I*_(*t*)+*Y*_*S*_(*t*)+*Y*_*I*_(*t*)+*F*_*S*_(*t*)+*F*_*I*_(*t*). In (20), the coefficients *B* and *C*_*i*_ are the positive weights associated with *S* and *u*_*i*_, respectively, showing the relative importance among them. We want to find optimal control triplet *u*^*∗*^=(*u*_1_^*∗*^, *u*_2_^*∗*^, *u*_3_^*∗*^)^*T*^, such that

(21) $$\begin{array}{c} J\left(u^{*}\right)=\underset{u\in U}{\max}J\left(u\right). \end{array}$$

The optimal control problem can loosely be stated as selecting a control law *u*(*t*) among all admissible controls in *𝕌* in (19) and corresponding state variables that maximize the performance index (20) and governs the system (2)–(12) from fixed initial states (13) to free terminal states:

(22) $$\begin{array}{c} L_{S}\left(T\right),L_{I}\left(T\right),M_{S}\left(T\right),M_{I}\left(T\right),Y_{S}\left(T\right),Y_{I}\left(T\right),F_{S}\left(T\right),F_{I}\left(T\right),S\left(T\right),I\left(T\right),Y_{f}\left(T\right)\text{ are all free}. \end{array}$$

Since we have bounded Lebesgue measurable controls in (19) and nonnegative initial conditions (13), then nonnegative bounded solutions to systems (2)–(12) exist. Besides, since the integrand in objective functional (21) is concave for *u* on the concave and closed admissible control set *𝕌* in (19), systems (2)–(12) are linear in the control variables *u*_*i*_, and the state variables are all bounded; then, the existence of optimal control *u*^*∗*^ is guaranteed [52], and the optimal *u*_*i*_^*∗*^ is guaranteed to maximize (21) based on the Mangasarian sufficient condition [53].

The first-order necessary conditions that an optimal triplet must satisfy are derived from Pontryagin's maximum principle [54]. This principle transforms the optimal control problem (21) with system constraints (2)–(12) into a problem of maximizing pointwise a Hamiltonian *H* for *u*_1_, *u*_2_, and *u*_3_. For the underlying control problem, the Hamiltonian *H* is given as follows:

(23) $$\begin{array}{c} H=-\;\left(C_{0}N+C_{1}u_{1}^{2}+C_{2}u_{2}^{2}+C_{3}u_{3}^{2}\right)+\sum_{i=1}^{11}p_{i}R_{i}, \end{array}$$

where *p*_*i*_(*i*=1,2,…, 11) is the adjoin function of time *t* corresponding to state variables and must be determined by the optimization process, and *R*_*i*_(*i*=1,2,…, 11) is the right-hand side of systems (2)–(12) in that order. The adjoin function *p*_*i*_ can be considered as the shadow price of the performance index (20) for the initial conditions. The optimality conditions according to Pontryagin's maximum principle are provided by the following three system blocks:

(24) $$\begin{array}{c} \frac{\partial H}{\partial u_{i}}=0,\;i=1,2,3, \end{array}$$

(25) $$\begin{array}{c} {\dot{x}}_{i}=\frac{\partial H}{\partial p_{i}},\;i=1,2,\ldots ,9,x_{i}\in X, \end{array}$$

(26) $$\begin{array}{c} {\dot{p}}_{i}=-\frac{\partial H}{\partial x_{i}},\;i=1,2,\ldots ,9,x_{i}\in X, \end{array}$$

where *X* is the vector of state variables, i.e., *x*_*i*_ ∈ {*L*_*S*_, *L*_*I*_, *M*_*S*_, *M*_*I*_, *Y*_*S*_, *Y*_*I*_, *F*_*S*_, *F*_*I*_, *S*, *I*, *Y*_*f*_}. We call the first block (24) the optimal controls, the second block (25) the dynamical systems, and the third block (26) the adjoint systems. Application of condition (25) provides the dynamical systems (2)–(12). The equations of optimal controls and the adjoint system are presented in the following theorems.

**Theorem 1 .**

The optimal controls *u*_1_^*∗*^, *u*_2_^*∗*^, and *u*_3_^*∗*^ that satisfy (24) are given by

(27) $$\begin{array}{c} u_{1}^{*}=\min\left\{1,\max\left\{0,-\frac{p_{1}\varepsilon_{L}L}{2C_{1}}\right\}\right\}, \end{array}$$

(28) $$\begin{array}{c} u_{2}^{*}=\min\left\{1,\max\left\{0,\frac{p_{11}Α}{2C_{2}}\right\}\right\}, \end{array}$$

(29) $$\begin{array}{c} u_{3}^{*}=\min\left\{1,\max\left\{0,-\frac{p_{10}\varepsilon_{I}P_{I}}{2C_{3}}\right\}\right\}. \end{array}$$

**Proof**

By applying (24), we have

(30) $$\begin{array}{c} \frac{\partial H}{\partial u_{1}}=0\Leftrightarrow u_{1}=-\frac{p_{1}\varepsilon_{1}L}{2C_{1}},\\ \frac{\partial H}{\partial u_{2}}=0\Leftrightarrow u_{2}=\frac{p_{11}Α}{2C_{2}},\\ \frac{\partial H}{\partial u_{3}}=0\Leftrightarrow u_{3}=-\frac{p_{10}\varepsilon_{I}P_{I}}{2C_{3}}. \end{array}$$

Expressions (27)–(29) are obtained by realizing that all control variables are bounded, i.e., 0 ≤ *u*_*i*_ ≤ 1 as imposed in (19). It means that if *u*_*i*_ < 0 for some interval of *t*, then we set *u*_*i*_=0 in that interval. Similarly, if *u*_*i*_ > 1 for some *t*, then we set *u*_*i*_=1. These expressions are useful in finding numerical solutions to the problem.

**Theorem 2 .**

There exists an optimal control triplet *u*_1_^*∗*^, *u*_2_^*∗*^, and *u*_3_^*∗*^ by (24) and corresponding optimal state variables *L*_*S*_^*∗*^, *L*_*I*_^*∗*^, *M*_*S*_^*∗*^, *M*_*I*_^*∗*^, *Y*_*S*_^*∗*^, *Y*_*I*_^*∗*^, *F*_*S*_^*∗*^, *F*_*I*_^*∗*^, *S*^*∗*^, *I*^*∗*^, and *Y*_*f*_^*∗*^ by (25) that satisfy *J*(*u*_1_^*∗*^, *u*_2_^*∗*^, *u*_3_^*∗*^)=max*J*(*u*_1_, *u*_2_, *u*_3_) in (21). Furthermore, from (26), there exist adjoint functions *p*_1_, *p*_2_,…, *p*_11_, such that

(31) $$\begin{array}{c} {\dot{p}}_{1}=C_{0}+p_{1}\left(\frac{b_{1}\left(F_{S}+F_{I}\right)}{c\left(m+S+I\right)}+v_{L}+d_{L}+\varepsilon_{1}u_{1}\right)+\frac{\left(p_{1}-p_{2}\right)\alpha_{2,L}I}{m+S+I}-\left(p_{3}\left(1-r\right)+p_{4}r\right)v_{L}+\frac{\left(p_{9}S+p_{10}I\right)\eta_{L}}{m+S+I},\\ {\dot{p}}_{2}=C_{0}+\frac{p_{1}b_{1}\left(F_{S}+F_{I}\right)}{c\left(m+S+I\right)}+p_{2}\left(v_{L}+d_{L}+\varepsilon_{1}u_{1}\right)-p_{4}\left(1-r\right)v_{L}-prv_{L}+\frac{p_{9}\left(\alpha_{1,L}+\eta_{L}\right)S-p_{10}\left(\alpha_{1,L}S-\eta_{L}I\right)}{m+S+I},\\ {\dot{p}}_{7}=C_{0}-p_{1}b_{1}\left(1-\frac{L_{S}+L_{I}}{c\left(m+S+I\right)}\right)+\left(p_{7}-p_{5}\right)\delta +p_{7}d_{F}+\frac{\left(p_{7}-p_{8}\right)\alpha_{2,Y}I}{m+S+I}+\frac{\left(p_{9}S+p_{10}I\right)\eta_{Y}}{m+S+I},\\ {\dot{p}}_{8}=C_{0}-p_{1}b_{1}\left(1-\frac{L_{S}+L_{I}}{c\left(m+S+I\right)}\right)+\left(p_{8}-p_{6}\right)\delta +p_{8}d_{F}+\frac{p_{9}\left(\alpha_{1,Y}+\eta_{Y}\right)S-p_{10}\left(\alpha_{1,Y}S-\eta_{Y}I\right)}{m+S+I},\\ {\dot{p}}_{9}=-B-\frac{p_{1}b_{1}\left(F_{S}+F_{I}\right)L_{S}}{c{\left(m+S+I\right)}^{2}}+\left(\left(p_{2}-p_{1}\right)\alpha_{2,L}L_{S}+\left(p_{4}-p_{3}\right)\alpha_{2,M}M_{S}+\alpha_{2,Y}\left(\left(p_{6}-p_{5}\right)Y_{S}+\left(p_{8}-p_{7}\right)F_{S}\right)\right)\frac{I}{{\left(m+S+I\right)}^{2}}\\ +p_{9}\left(\frac{b_{2}\left(2S+I\right)}{K}+\frac{\left(m+I\right)\left(V_{\alpha }+N_{\eta }\right)}{{\left(m+S+I\right)}^{2}}+\frac{\alpha_{3}I^{2}}{{\left(S+I\right)}^{2}}+d_{S}\right)\\ -p_{10}\left(\frac{\left(m+I\right)V_{\alpha }}{{\left(m+S+I\right)}^{2}}+\frac{\alpha_{3}I^{2}}{{\left(S+I\right)}^{2}}+\frac{N_{\eta }I}{{\left(m+S+I\right)}^{2}}\right),\\ {\dot{p}}_{10}=-\frac{p_{1}b_{1}\left(F_{S}+F_{I}\right)L}{c{\left(m+S+I\right)}^{2}}+\left(\left(p_{3}-p_{4}\right)\alpha_{2,L}L_{S}+\left(p_{3}-p_{4}\right)\alpha_{2,M}M_{S}+\alpha_{2,Y}\left(\left(p_{5}-p_{4M6}\right)Y_{S}+\left(p_{7}-p_{8}\right)F_{S}\right)\right)\\ \frac{m+S}{{\left(m+S+I\right)}^{2}}+p_{9}\left(\frac{b_{2}}{K}-\frac{\left(V_{\alpha }+N_{\eta }\right)S}{{\left(m+S+I\right)}^{2}}+\frac{\alpha_{3}S^{2}}{{\left(S+I\right)}^{2}}\right)\\ +p_{10}\left(\frac{V_{\alpha }S}{{\left(m+S+I\right)}^{2}}-\frac{\alpha_{3}S^{2}}{{\left(S+I\right)}^{2}}+\frac{\left(m+S\right)N_{\eta }}{{\left(m+S+I\right)}^{2}}+d_{I}+\varepsilon_{3}u_{3}\right). \end{array}$$

The equations for ${\dot{p}}_{3}$, ${\dot{p}}_{4}$, ${\dot{p}}_{5}$, and ${\dot{p}}_{6}$ are depending on *ρ* in (1). If *γ*(*M*_*S*_+*M*_*I*_) < *Y*_*S*_+*Y*_*I*_+*Y*_*f*_ (male scarcity), then we have

(32) $$\begin{array}{c} {\dot{p}}_{3}=C_{0}+p_{3}d_{M}+\frac{\left(p_{3}-p_{4}\right)\alpha_{2,M}I+\left(p_{9}S+p_{10}I\right)\eta_{M}}{m+S+I}+\frac{\left(\left(p_{5}-p_{7}\right)Y_{S}+\left(p_{6}-p_{8}\right)Y_{I}\right)v_{Y}\gamma }{Y_{S}+Y_{I}+Y_{f}}, \end{array}$$

(33) $$\begin{array}{c} {\dot{p}}_{4}=C_{0}+p_{4}d_{M}+\frac{\left(\left(p_{5}-p_{7}\right)Y_{S}+\left(p_{6}-p_{8}\right)Y_{I}\right)v_{Y}\gamma }{Y_{S}+Y_{I}+Y_{f}}+\frac{\left(p_{9}\left(\alpha_{1,M}+\eta_{M}\right)S-p_{9}\left(\alpha_{1,M}S-\eta_{M}I\right)\right)}{m+S+I}, \end{array}$$

(34) $$\begin{array}{c} {\dot{p}}_{5}=C_{0}+p_{5}d_{Y}+\frac{\left(\left(p_{5}-p_{7}\right)\left(Y_{I}+Y_{f}\right)+\left(p_{8}-p_{6}\right)Y_{I}\right)v_{Y}\gamma \left(M_{S}+M_{I}\right)}{{\left(Y_{S}+Y_{I}+Y_{f}\right)}^{2}}+\frac{\left(p_{5}-p_{6}\right)\alpha_{2,Y}I+\left(p_{9}S+p_{10}I\right)\eta_{Y}}{m+S+I}, \end{array}$$

(35) $$\begin{array}{c} {\dot{p}}_{6}=C_{0}+p_{6}d_{Y}+\frac{\left(\left(p_{7}-p_{5}\right)Y_{S}+\left(p_{6}-p_{8}\right)\left(Y_{S}+Y_{f}\right)\right)v_{Y}\gamma \left(M_{S}+M_{I}\right)}{{\left(Y_{S}+Y_{I}+Y_{f}\right)}^{2}}+\frac{\left(p_{9}\left(\alpha_{1,Y}+\eta_{Y}\right)S-p_{10}\left(\alpha_{1,Y}S-\eta_{Y}I\right)\right)}{m+S+I}, \end{array}$$

(36) $$\begin{array}{c} {\dot{p}}_{11}=\frac{\left(\left(p_{7}-p_{5}\right)Y_{S}+\left(p_{8}-p_{6}\right)Y_{I}\right)v_{Y}\gamma \left(M_{S}+M_{I}\right)}{{\left(Y_{S}+Y_{I}+Y_{f}\right)}^{2}}+p_{11}\varphi . \end{array}$$

Otherwise, if *γ*(*M*_*S*_+*M*_*I*_) ≥ *Y*_*S*_+*Y*_*I*_+*Y*_*f*_ (male abundance), then we get

(37) $$\begin{array}{c} {\dot{p}}_{3}=C_{0}+p_{3}d_{M}+\frac{\left(p_{3}-p_{4}\right)\alpha_{2,Y}I+\left(p_{9}S+p_{10}I\right)\eta_{Y}}{m+S+I}, \end{array}$$

(38) $$\begin{array}{c} {\dot{p}}_{4}=C_{0}+p_{4}d_{M}+\frac{\left(p_{9}\left(\alpha_{1,Y}+\eta_{Y}\right)S-p_{10}\left(\alpha_{1,Y}S-\eta_{Y}I\right)\right)}{m+S+I}, \end{array}$$

(39) $$\begin{array}{c} {\dot{p}}_{5}=C_{0}+p_{5}d_{Y}+\left(p_{5}-p_{7}\right)v_{Y}+\frac{\left(p_{5}-p_{6}\right)\alpha_{2,Y}I+\left(p_{9}S+p_{10}I\right)\eta_{Y}}{m+S+I}, \end{array}$$

(40) $$\begin{array}{c} {\dot{p}}_{6}=C_{0}+p_{6}d_{Y}+\left(p_{6}-p_{8}\right)v_{Y}+\frac{\left(p_{9}\left(\alpha_{1,Y}+\eta_{Y}\right)S-p_{10}\left(\alpha_{1,Y}S-\eta_{Y}I\right)\right)}{m+S+I}, \end{array}$$

(41) $$\begin{array}{c} {\dot{p}}_{11}=p_{11}\varphi . \end{array}$$

The following transversality conditions must be fulfilled:

(42) $$\begin{array}{c} p_{9}\left(T\right)=B,p_{i}\left(T\right)=0,\;\text{for }i\neq 9. \end{array}$$

**Proof**

When applying condition (26), use (32)–(36) for male scarce case and use (37)–(41) for male abundance case. Transversality condition (42) must be imposed as we assume free terminal times with scrap function.

## 5. Numerical Simulations and Discussion

We investigate the effects of control measures in controlling the dynamics by considering the case of grapevine leaf-roll associated virus (GLRaV) spread by *Planococcus ficus.* This insect also damages the plants as it excretes a large amount of honeydew. We used the values of parameters presented in Table 1 mainly taken from [55–57]. Furthermore, for the plant growth rate, we used the after bloom fruit growth rate from the Pinot noir simulation conducted in [58]. The selection of *γ* also considers the fact of possible multiple mating of *Planococcus ficus* [59]. In this simulation, we also assume that initially, the sex ratio is highly male-biased, and thus, *γ*(*M*_*S*_+*M*_*I*_) ≥ *Y*_*S*_+*Y*_*I*_+*Y*_*f*_. We weigh the terms in functional objective by *B*=1 and *C*_0_=1, while *C*_1_=*C*_2_=*C*_3_=50 with *T*=80 days as the length of the control period.

### 5.1. Model with and without Control

The control problem is numerically solved by the forward-backward sweep method [60] in combination with the well-known fourth-order Runge–Kutta algorithm. To investigate the overall effect of the control mix in more detail, we consider four strategies as presented in Table 2, where of three control instruments available, we implement at least two of them. By strategy A, we apply all available controls *u*_1_, *u*_2_, and *u*_3_ to achieve control objective, and by strategies B, C, and D, we employ only two controls of different combinations. In this current setting, a no-control strategy is a situation where there is no-control measure applied, i.e., we set *u*_1_=*u*_2_=*u*_3_=0.

#### 5.1.1. Infectious Insects

**Figure 2:**
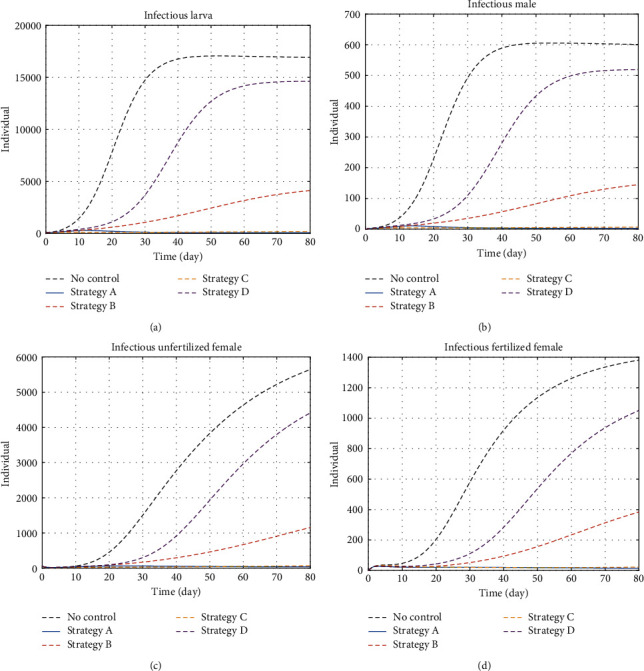
The dynamics of infectious larvae and infectious adult insects under control and no-control strategies. (a) Larvae, (b) infectious males, (c) infectious unfertilised females, and (d) infectious fertilised females. The dashed black line stands for no-control strategy.

All infectious insect dynamics have a quite similar behaviour (Figure 2). Without control, the infectious insect population grows exponentially until a certain amount of population. The infectious larva population grows from 100 insects to above 15,000 in 40 days. This is due to the high number of eggs that are laid by a single female, and the highest transmission rate of pathogen happens during the larval stage. Strategy D manages to reduce the population of the infectious larva to be below 15,000 insects. However, this strategy does not significantly reduce the number of infectious larvae because it does not directly kill insects. Comparatively, strategy C manages to reduce the infectious larva population significantly. Strategy B in the other hand manages to reduce the infectious larva population even when it does not exploits plant removal as it kills larvae with green insecticide.

#### 5.1.2. Infected and Susceptible Plants

**Figure 3:**
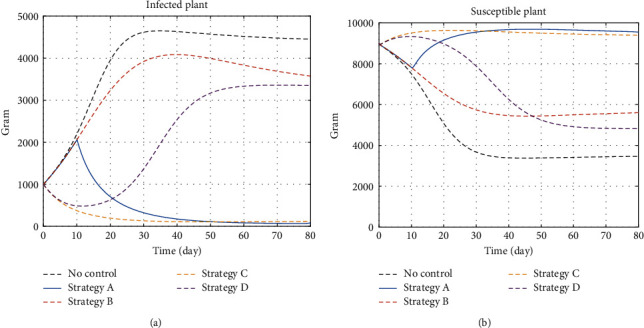
The dynamics of infected and susceptible plants under control and no-control strategies. (a) Infected plants and (b) susceptible plants. Without intervention, the size of the susceptible plant significantly decreases and the infected plant significantly increases.

Compared to the ideal condition, we face 65.36% of loss without control.  Figure 3(b) shows that susceptible plant mass drops to 3.46 kg when it is supposed to grow to the maximum carrying capacity which is 10 kg. Strategy A manages to reduce the loss percentage to only 1.61%. This is expected because strategy A exploits all control variables to prevent physical damage and pathogen transmission. Strategy C manages to reduce the loss to 6.12% by killing the larva and removing infectious plants. This makes sense as the larvae of *Planococcus ficus* is more destructive than the adults. Interesting results occur in strategy B and strategy D. For the first half of the control period, strategy D shows the sign that it reduces the potential loss more than strategy B. In the end, strategy B generates more yields. It is because mating disruption delays the production of larva, while it does not actively kill the existing larvae, with the fact that the infectious plant population remains regardless of the control effort, and thus in strategy D, the population of larva still increases and larva can still become an infectious larva.

The size of the infected plant increases significantly without control even when the initial infectious insect population is low. Strategy B that actively controls the insect behaviours without removing infected plants manages to reduce the amount of infected plant by reducing the vector of the pathogen. Strategy D shows a quite similar result where the amount of infected plant increases. Different from strategy B, strategy D does not actively kill larva, and thus, the density of infected plant increases. Strategy A and strategy C manage to decrease infected plant significantly because they actively kill larva and remove the infectious plant with slightly lower infected plant density generated by strategy A.

#### 5.1.3. Susceptible Insects

**Figure 4:**
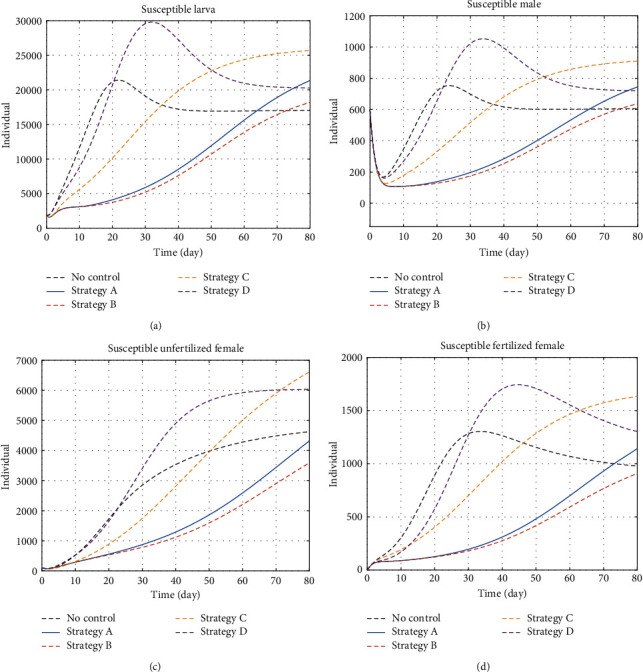
The dynamics of susceptible insects under control and no-control strategies. (a) Susceptible larva, (b) susceptible males, (c) susceptible of unfertilised females, and (d) susceptible of fertilised females. The control effects vary among strategies.

It was confirmed that the active control to insects applying green insecticides and mating disruption reduces the amount of insect population. Strategy A shows a higher susceptible larva population than the result of strategy B because strategy A also removes the infected plant and reduces the possibility of the plant to insect pathogen transmission. Strategy C and strategy D show a higher susceptible insect population. There are two factors to this; first is that both strategy C and strategy D only exploit one control effort to reduce the insect population. The second is that both strategies actively remove infected plant and prevent the plant from insect pathogen transmission. On the last day, the susceptible insect population generated by strategy C is higher than strategy D because the amount of infectious insect is higher in strategy D (Figure 4).

#### 5.1.4. Male-to-Female Ratio and Probability of Mating

**Figure 5:**
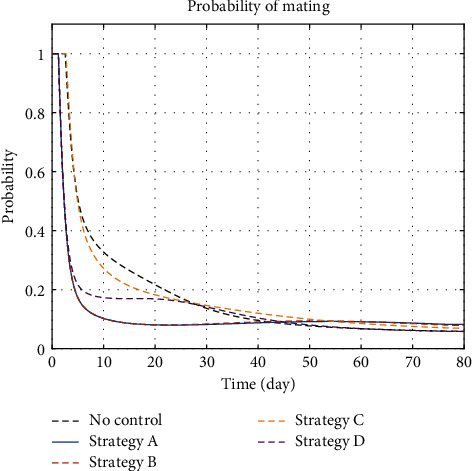
The dynamical systems of insect-plant interaction are highly influenced by sex ratio. Parameter *ρ* determines the situation of male abundance and male scarcity.

The insect-plant interaction is alternating between male abundance and male scarce. In the case of male abundance, the probability of mating between a male and an unfertilised female is equal to 1, and in the case of male scarce, the probability of mating is less than 1.  Figure 5 describes the male-to-female ratio for strategies. It was that any strategy with mating disruption, i.e., strategies A, B, and D, accelerates the switching time between male abundance and male scarce. The case of polyandry also exists among mealybugs where female insects can mate with multiple males and further make control efforts to insect population that becomes more effective as the probability of mating tends to be lower than one. However, this is not always the case. In [36], the insect used as the example is the fruit fly *Bactrocera invadens* where the male can mate with multiple females. This makes the switching to male scarce that requires greater effort.

#### 5.1.5. The Optimal Controls

**Figure 6:**
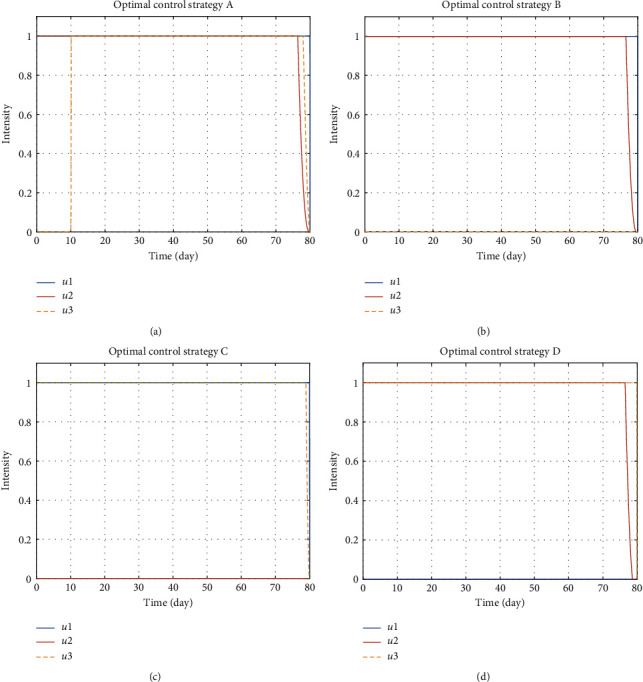
The evaluated three control measures, namely, the use of green insecticide *u*_1_, the application of mating disruption *u*_2_, and the removal of infected plants *u*_3_, under four different control strategies. (a) Strategy A used all measures. (b) Strategy B applied *u*_1_ and *u*_2_. (c) Strategy C used *u*_1_ and *u*_3_. (d) Strategy D occupied *u*_2_ and *u*_3_. Each strategy requires different implementations.

Finding optimal controls is the main task in this study.  Figure 6 illustrates the optimal controls which maximize the performance index under different intervention strategies. Whenever applicable, green insecticides should be fully applied during the whole control period. This indicates the importance of the green insecticides when it is applicable even when the effectiveness of such control is relatively low. Strategy A suggests that mating disruption should be implemented fully up to around day 76 of the control period, while plant removal starts on day 10 of the control period up to around day 78 of the control period (Figure 6(a)). Meanwhile, strategy B recommends quite similar control applications with strategy A, while plant removal is not conducted (Figure 6(b)). By strategy C, the use of green insecticide is fully implemented for the whole control period. With the absence of mating disruption, plant removal should be implemented fully from the beginning of the control period up to around day 78 of the control period (Figure 6(c)). By strategy D, the mating disruption is fully carried from the beginning of the control period up until day 77 of the control period (Figure 6(d)).

### 5.2. Cost-Effectiveness Analysis

To compare the strategies presented, two cost-effectiveness metrics are utilized, namely, the incremental cost-effectiveness ratio (ICER) and the average cost-effectiveness ratio (ACER). ICER can loosely be defined as the incremental cost per incremental benefit. In health, ICER is an incremental ratio of the difference in total incurred cost between one strategy and the next best strategy to the difference in the total number of averted infections through each strategy [61, 62]. ACER is the ratio between cost and benefit, i.e., it can be the ratio between the total cost incurred by intervention and the total infection averted [63]. In this work, the outcome is represented by the total growth of healthy plants managed by each strategy. Let *G*_*i*_ denotes the total growth managed by strategy *i* during the control period [0, *T*]; then, *G*_*i*_ is calculated as

(43) $$\begin{array}{c} G_{i}=B\left(S^{i}\left(T\right)-\bar{S}\left(T\right)\right). \end{array}$$

In (43), *S*^*i*^ is the optimal size of healthy plants obtained by strategy *i*, where *i* ∈ {*A*, *B*, *C*, *D*}, and *S* is the size of healthy plants under a no-control strategy as provided in Figure 3(b). The total control cost *Q*_*i*_ of strategy *i* during the control period [0, *T*] is given by

(44) $$\begin{array}{c} Q_{i}=\int_{0}^{T}\left(c_{1}u_{1,i}^{2}\left(t\right)+c_{2}u_{2,i}^{2}\left(t\right)+c_{3}u_{3,i}^{2}\left(t\right)\right)\text{d}t, \end{array}$$

where *u*_1,*i*_, *u*_2,*i*_, and *u*_3,*i*_ are the optimal controls obtained by strategy *i*, where *i* ∈ {*A*, *B*, *C*, *D*}, as shown in Figure 5. Note that, based on Table 2, we have *u*_3,*B*_=0, *u*_2,*C*_=0, and *u*_1,*D*_=0. ICER and ACER are then calculated by using the following formulae:

(45) $$\begin{array}{c} \text{ICER}\left(k\right)=\frac{Q_{k}-Q_{k-1}}{G_{k}-G_{k-1}}, \end{array}$$

(46) $$\begin{array}{c} \text{ACER}\left(k\right)=\frac{Q_{k}}{G_{k}}, \end{array}$$

for *k*=1,2,3,4. In the case of ICER calculation, the strategies must be ascendingly ordered according to the total growth managed as shown in Table 3, where *G*_0_ and *Q*_0_ refer to the total growth managed and the total cost incurred by a no-control policy which is both zero. By (45) and (46), the smaller the ICER and ACER values, the more cost-effective the strategy. Based on ACERs, it is suggested that strategy B is the most cost-effective followed by strategy A. However, the calculation of ICERs for more than two strategies requires more steps.

The ranking in Table 3 shows that strategy D contributed the least quantity of plant growth and strategy A provided the most quantity of growth. We calculate initial estimates of the incremental cost, incremental plant growth, and ICERs between consecutive pairs of nondominated strategies. Based on Table 3, we concluded that due to its smaller value of ICER, strategy C is the most cost-effective strategy in intensifying the size of healthy plants under minimal control efforts. This is also consistent with the result from ACER calculations where strategy C becomes the most cost-effective strategy, while strategy A becomes the second best strategy.

**Figure 7:**
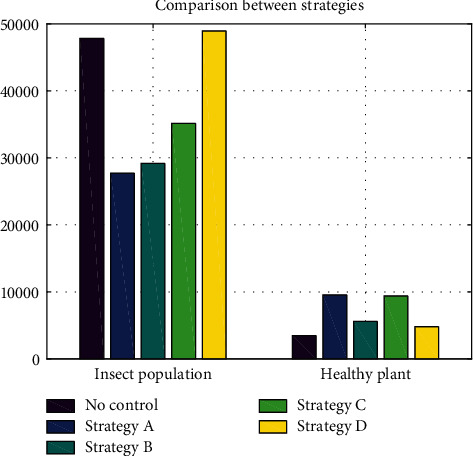
The comparison of the total insect population and healthy plant between all strategies at the terminal time.

As the most cost-effective control scenario, strategy C successfully reduces the potential loss to only 6.12%. Consequently, this strategy increases the mass of healthy plants by 5.92 kg. The decrease of potential loss is 1.61% from the result of strategy A which is the strategy that generates the most benefit and the second-best strategy based on its ACER value. The comparison between all strategies is shown in Figure 7. It was observed that even though strategy B is effective in reducing the total insect population, the size of the healthy plant it generates is not that high. This indicates that both insect population control and plant removal are needed to generate the most benefit. The fact that strategy C becomes the best strategy indicates that the decision to use what control to control what population is also an important consideration. In this case, using green insecticide and conducting plant removal is enough to be the most cost-effective even if it does not generate the most benefit.

## 6. Conclusion

In this work, we proposed a model of plant-insect interaction governed by a set of nonlinear ordinary differential equations outfitted with three control variables, namely, green insecticides, mating disruption, and infected plant removal. Using an optimal control approach and Pontryagin's maximum principle, we provided the analytical framework of the existence of the optimal set of controls and with Mangasarian condition that makes Pontryagin's maximum principle to become the necessary and sufficient optimality condition. We explored and simulated four control strategies incorporating different combinations of controls consisting of the combination of three controls and the combinations of two controls with the study case of *Planococcus ficus* and GLRaV. We further conducted a cost-effectiveness analysis to compare each strategy. The cost-effectiveness analysis recommends an optimal control mix without mating disruption (strategy C), while the combination of all three controls (strategy A) is the second most cost-effective strategy.

From the previous sections, we observed that all strategies managed to decrease the pest insect population and increase the density of a healthy plant. The most benefit is generated by strategy A as it exploits all three control measures, while strategy C is the second most beneficial strategy. The green insecticide in this work directly attacks the larvae population. This significantly reduces the larvae population compared to the method of mating disruption. However, the release of synthetic sex pheromones also hindered the mate finding between a male and unfertilised females. This intervention prevents the transmission flow from unfertilised compartment to fertilised one, resulting in a decline of eggs deposit and larvae population.

The first two controls deals with the insect population, and the third control deals directly with the removal of infected plants. Even if there are many examples where plants removal techniques have been successfully implemented but many where they have not [37], the integration between insect population control and plant removal is needed as it deals with both ends of the control efforts which are to reduce insects that feed on plant and spread diseases and at the same time preventing insects and another part of the plant to be infected. It has been revealed in this study that infected plants removal, even with low effectiveness, is still an important control method.

Regardless of the benefits that the optimum control framework offers in evaluating several pest control scenarios, this theoretical study needs validation through a field experiment to verify the model and parameters.

## Figures and Tables

**Table 1:** Parameters and control variables of the model.

Parameter	Description	Unit	Value
*L* ^0^	The initial size of the susceptible larvae	Individual	1900
*L*_*v*_^0^	The initial size of the infectious larvae	Individual	100
*M* ^0^	The initial size of the noninfectious male insects	Individual	100
*M*_*v*_^0^	The initial size of the infectious male insect	Individual	50
*Y* ^0^	The initial size of noninfectious unfertilised female insects	Individual	0
*Y*_*v*_^0^	The initial size of infectious unfertilised female insects	Individual	0
*Y*_*f*_^0^	The initial size of fake female insects	Individual	0
*F* ^0^	The initial size of the noninfectious fertilised female insects	Individual	600
*F*_*v*_^0^	The initial size of infectious fertilised female insects	Individual	0
*S* ^0^	The initial amount of susceptible plants	Gram	9000
*I* ^0^	The initial amount of infected plants	Gram	1000
*A*	Maximum synthetic sex pheromone deployed in a day	Individual/day	50
*ρ* ^0^	The initial mating capacity	−	2
*b*_1_	The intrinsic egg-laying rate	1/day	10.4
*b*_2_	The intrinsic plant growth	Gram/day	3.842
*K*	The carrying capacity for plant	Gram	10000
*r*	The proportion of female-to-male insect population from larvae	−	0.4
*v*_*L*_	The rate of transfer from larva to unfertilised female insect	1/day	1/28.05
*v*_*Y*_	The rate of mating for unfertilised female	1/day	0.5
*m*	The constant of half-saturation	Gram	0.8
*δ*	The rate of transfer from fertilised female to unfertilised female	1/day	0.1
*α*_1,*M*_	The transmission rate of the pathogen from infected plants to susceptible adult males	(Gram/individual)/day	0
*α*_1,*Y*_	The transmission rate of the pathogen from infected plants to susceptible adult females	(Gram/individual)/day	0.01
*α*_1,*L*_	The transmission rate of the pathogen from infected plants to susceptible larva	(Gram/individual)/day	0.18
*α*_2,*M*_	The transmission possibility of the pathogen from infectious adult males to susceptible plants during consumption	1/day	0
*α*_2,*Y*_	The transmission possibility of the pathogen from infectious adult females to susceptible plants during consumption	1/day	0.01
*α*_2,*L*_	The transmission possibility of the pathogen from infectious larva to susceptible plants during consumption	1/day	0.18
*η*_*M*_	Consumption rate of adult males	(Gram/individual)/day	0
*η*_*Y*_	Destruction rate of adult females	(Gram/individual)/day	0.05
*η*_*L*_	Destruction rate of larva	(Gram/individual)/day	0.05
*α*_3_	The transmission rate of the pathogen from infectious plant to susceptible plants	1/day	0.2
*γ*	The number of females that can be fertilised by a single male	Individual/day	0.5
*c*	The number of larvae that can live within support of one unit of crop	Individual/gram	5
*d*_*L*_	The natural mortality rate of larvae	1/day	1/15
*d*_*M*_	The natural mortality rate of the male insect	1/day	1/1.66
*d*_*Y*_	The natural mortality rate of the unfertilised female insect	1/day	1/27.64
*d*_*F*_	The natural mortality rate of the fertilised female insect	1/day	1/27.64
*d*_*S*_	The natural mortality rate of susceptible plant	1/day	0
*d*_*I*_	The natural mortality rate of infected plant	1/day	0.1
*φ*	The fading rate of synthetic sex pheromone	1/day	1/6
*ε*_*L*_	The effectiveness of control *u*_1_	−	0.2
*ε*_*I*_	The effectiveness of control *u*_3_	−	0.2
Control variable	Description	Unit	Value
*u*_1_(*t*)	The proportion of larva population that green insecticide is applied to at time *t*	−	(0,1)
*u*_2_(*t*)	The proportion of the maximum synthetic sex pheromone deployed at time *t*	−	(0,1)
*u*_3_(*t*)	The proportion of infected plant that will be removed at time *t*	−	(0,1)

**Table 2:** Control strategies.

Strategy	Control action		
	*u*_1_	*u*_2_	*u*_3_
No control	0	0	0
A	(0,1)	(0,1)	(0,1)
B	(0,1)	(0,1)	0
C	(0,1)	0	(0,1)
D	0	(0,1)	(0,1)

**Table 3:** Calculation of ACER and ICER.

*k*	Strategy	Total growth managed	Cost	ACER	ICER	
0	No control	0	0	NA	NA	NA
1	D	1355.1823	783.0009	0.577783	0.577783	ed
2	B	2140.011	784.8544	0.366753	d	d
3	C	5923.3194	795.7232	0.134337	0.002785	0.134337
4	A	6084.0714	1126.946	0.185229	2.06046	2.06046

d, dominated; ed, extendedly dominated.

## Data Availability

The data used to support the findings of this study are included within the article.
